# Association of Functional Polymorphisms in *Interferon Regulatory Factor 2* (*IRF2*) with Susceptibility to Systemic Lupus Erythematosus: A Case-Control Association Study

**DOI:** 10.1371/journal.pone.0109764

**Published:** 2014-10-06

**Authors:** Aya Kawasaki, Hiroshi Furukawa, Nao Nishida, Eiji Warabi, Yuya Kondo, Satoshi Ito, Isao Matsumoto, Makio Kusaoi, Hirofumi Amano, Akiko Suda, Shouhei Nagaoka, Keigo Setoguchi, Tatsuo Nagai, Shunsei Hirohata, Kota Shimada, Shoji Sugii, Akira Okamoto, Noriyuki Chiba, Eiichi Suematsu, Shigeru Ohno, Masao Katayama, Akiko Okamoto, Hajime Kono, Katsushi Tokunaga, Yoshinari Takasaki, Hiroshi Hashimoto, Takayuki Sumida, Shigeto Tohma, Naoyuki Tsuchiya

**Affiliations:** 1 Molecular and Genetic Epidemiology Laboratory, Faculty of Medicine, University of Tsukuba, Tsukuba, Ibaraki, Japan; 2 Clinical Research Center for Allergy and Rheumatology, Sagamihara Hospital, National Hospital Organization, Sagamihara, Kanagawa, Japan; 3 Research Center for Hepatitis and Immunology, National Center for Global Health and Medicine, Ichikawa, Chiba, Japan; 4 Environmental Molecular Biology Laboratory, Faculty of Medicine, University of Tsukuba, Tsukuba, Ibaraki, Japan; 5 Department of Internal Medicine, Faculty of Medicine, University of Tsukuba, Tsukuba, Ibaraki, Japan; 6 Department of Rheumatology, Niigata Rheumatic Center, Shibata, Niigata, Japan; 7 Department of Internal Medicine and Rheumatology, Juntendo University School of Medicine, Tokyo, Japan; 8 Department of Rheumatology, Yokohama Minami Kyosai Hospital, Yokohama, Kanagawa, Japan; 9 Center for Rheumatic Diseases, Yokohama City University Medical Center, Yokohama, Kanagawa, Japan; 10 Allergy and Immunological Diseases, Tokyo Metropolitan Cancer and Infectious Diseases Center Komagome Hospital, Tokyo, Japan; 11 Department of Rheumatology and Infectious Diseases, Kitasato University School of Medicine, Sagamihara, Kanagawa, Japan; 12 Department of Rheumatology, Tokyo Metropolitan Tama Medical Center, Fuchu, Tokyo, Japan; 13 Department of Rheumatology, Himeji Medical Center, National Hospital Organization, Himeji, Hyogo, Japan; 14 Department of Rheumatology, Morioka Hospital, National Hospital Organization, Morioka, Iwate, Japan; 15 Department of Internal Medicine and Rheumatology, Clinical Research Institute, Kyushu Medical Center, National Hospital Organization, Fukuoka, Japan; 16 Department of Internal Medicine, Nagoya Medical Center, National Hospital Organization, Nagoya, Aichi, Japan; 17 Department of Internal Medicine, Teikyo University School of Medicine, Tokyo, Japan; 18 Department of Human Genetics, Graduate School of Medicine, University of Tokyo, Tokyo, Japan; 19 Juntendo University School of Medicine, Tokyo, Japan; Oklahoma Medical Research Foundation, United States of America

## Abstract

Interferon regulatory factor 2 (IRF2) negatively regulates type I interferon (IFN) responses, while it plays a role in induction of Th1 differentiation. Previous linkage and association studies in European-American populations suggested genetic role of *IRF2* in systemic lupus erythematosus (SLE); however, this observation has not yet been confirmed. No studies have been reported in the Asian populations. Here we investigated whether *IRF2* polymorphisms contribute to susceptibility to SLE in a Japanese population. Association study of 46 *IRF2* tag single nucleotide polymorphisms (SNPs) detected association of an intronic SNP, rs13146124, with SLE. When the association was analyzed in 834 Japanese patients with SLE and 817 healthy controls, rs13146124 T was significantly increased in SLE compared with healthy controls (dominant model, P = 5.4×10^−4^, Bonferroni-corrected P [Pc] = 0.026, odds ratio [OR] 1.48, 95% confidence interval [CI] 1.18–1.85). To find causal SNPs, resequencing was performed by next-generation sequencing. Twelve polymorphisms in linkage disequilibrium with rs13146124 (r^2^: 0.30–1.00) were identified, among which significant association was observed for rs66801661 (allele model, P = 7.7×10^−4^, Pc = 0.037, OR 1.53, 95%CI 1.19–1.96) and rs62339994 (dominant model, P = 9.0×10^−4^, Pc = 0.043, OR 1.46, 95%CI 1.17–1.82). The haplotype carrying both of the risk alleles (rs66801661A–rs62339994A) was significantly increased in SLE (P = 9.9×10^−4^), while the haplotype constituted by both of the non-risk alleles (rs66801661G–rs62339994G) was decreased (P = 0.0020). A reporter assay was carried out to examine the effect of the *IRF2* haplotypes on the transcriptional activity, and association of the *IRF2* risk haplotype with higher transcriptional activity was detected in Jurkat T cells under IFNγ stimulation (Tukey's test, P = 1.2×10^−4^). In conclusion, our observations supported the association of *IRF2* with susceptibility to SLE, and the risk haplotype was suggested to be associated with transcriptional activation of *IRF2*.

## Introduction

Genome-wide association studies (GWAS) as well as candidate gene studies of systemic lupus erythematosus (SLE) have identified more than 70 susceptibility loci. However, they account for a small fraction of heritability of SLE; thus it appears that many susceptibility genes remain to be identified [1]–[11].

Interferon regulatory factor (IRF) family participates in induction of type I interferons (IFNs) and IFN-inducible genes [12]. IFNs play a crucial role in the pathogenesis of SLE [13], [14]. IRF family comprises of nine members, IRF1 to IRF9. IRFs have a conserved DNA binding domain with five tryptophan residues in the N-terminal region, and an IRF association domain (IAD) 1 or IAD2 in the C-terminal region, which mediates interactions with IRF members or other transcription factors [12]. Recent genetic studies have reported association of *IRF5*, *IRF7* and *IRF8* with SLE [1]–[3], [5], [6], [15], [16]. Especially, *IRF5* has already been established as an SLE susceptibility gene in various populations, including Japanese [17].

IRF2 is thought to negatively regulate type I IFN signals by competing with IRF1 for binding to the regulatory region of IFN and IFN-inducible genes [12]. In addition, IRF2 has a role in induction of Th1 differentiation [12], [18]. A recent study reported an association of *IRF2* with atopic dermatitis and eczema herpeticum [19]. With respect to SLE, Ramos et al. identified linkage of chromosome 4q34.3–35.1 to anti-Ro and/or La antibodies by linkage analysis in a European-American population [20]. This region included *IRF2* gene. They subsequently reported association of *IRF2* SNPs with SLE and dermatological manifestations in a family-based association study in a European-American population [21]. However, no replication studies have been published in populations of European descent. In addition, no studies have been published from Asian populations.

In the present study, we conducted a systematic association study to examine whether *IRF2* may contribute to genetic predisposition to SLE in a Japanese population. Our observations suggested that *IRF2* is associated with SLE, and the risk haplotype is associated with transcriptional activation of *IRF2*.

## Materials and Methods

### Patients and healthy controls

A total of 834 patients with SLE (66 males and 768 females, mean ± SD 45.3±15.0 years) and 817 healthy individuals (298 males and 519 females, 36.4±11.5 years) were studied. The patients and healthy individuals, all unrelated Japanese, were recruited at University of Tsukuba, Juntendo University, Sagamihara National Hospital, Tokyo Metropolitan Cancer and Infectious Diseases Center Komagome Hospital, Kitasato University, Yokohama Minami Kyosai Hospital, Tokyo Metropolitan Tama Medical Center, Himeji Medical Center, Kyushu Medical Center, Morioka Hospital, Teikyo University, Yokohama City University Medical Center, Nagoya Medical Center and the University of Tokyo. Among the healthy control genomic DNA, 266 were purchased from the Health Science Research Resources Bank. The patients fulfilled the American College of Rheumatology (ACR) criteria for SLE [22]. Presence or absence of renal disorder was also classified based on the ACR criteria.

### Ethics Statement

This study was reviewed and approved by University of Tsukuba Faculty of Medicine Research Ethics Committee, Sagamihara Hospital Research Ethics Committee, the University of Tokyo Research Ethics Committee, Juntendo University Research Ethics Committee, Yokohama Minami Kyosai Hospital Research Ethics Committee, Yokohama City University Medical Center Research Ethics Committee, Komagome Hospital Research Ethics Committee, Kitasato University Research Ethics Committee, Tama Medical Center Research Ethics Committee, Himeji Medical Center Research Ethics Committee, Morioka Hospital Research Ethics Committee, Kyushu Medical Center Research Ethics Committee, Nagoya Medical Center Research Ethics Committee, and Teikyo University Research Ethics Committee. This study was conducted according to the principles expressed in the Declaration of Helsinki. Written informed consent was obtained from all participants.

### Genotyping

Forty six tag single nucleotide polymorphisms (SNPs) in the *IRF2* region with minor allele frequency ≥0.05 were selected based on genotype and linkage disequilibrium (LD) data in the JPT (Japanese in Tokyo, Japan) on the HapMap Phase II+III data (http://hapmap.ncbi.nlm.nih.gov/), with *r^2^* threshold of 0.8.

Forty four of the tag SNPs were genotyped using the DigiTag2 assay as previously described [23]. The remaining two tag SNPs (rs793801 and rs3756093) were genotyped using the TaqMan SNP genotyping assays (Applied Biosystems, Foster City, CA). A pre-designed probe was used for rs3756093 (Assay ID: C__27512141_10), and custom probe for rs793801.

The association study was conducted in two stages, Firstly, association of the 46 tag SNPs was examined in the “discovery set”, which comprises of 501 SLE and 551 controls. Then the most significantly associated SNP, rs13146124, were genotyped in the remainder of the cases and controls, and two other SNPs detected by resequencing (rs66801661 and rs62339994) were genotyped in all 834 SLE and 817 healthy controls. Then the association of these three SNPs were examined in all cases and controls. A flow diagram is shown in Figure S1.

The SNPs rs66801661 and rs62339994 were genotyped using custom probes by TaqMan SNP genotyping assays.

### Resequencing of *IRF2*

Genomic DNA sequence around the *IRF2* gene was obtained from the NCBI database (Accession Number: NC_000004). All exons, the promoter region up to 5 kb upstream, and the intron 1 region of *IRF2* encompassing rs13146124 were captured using PCR. The *IRF2* region was amplified from genomic DNA of 12 individuals (six with rs13146124T/T and six with rs13146124C/C genotype) using nine primer pairs (Table S1). The nine amplicons from each individual were pooled, sheared using Covaris S220 (Covaris, Inc., Woburn, MA), and subjected to the 454 sequencing library preparation (Roche Applied Science, Indianapolis, IN). The libraries from the 12 individuals, tagged by distinct Multiplex Identifiers adaptors, were pooled. Subsequently, emulsion PCR and sequencing were carried out using the GS Junior System (Roche Applied Science). Analysis of the sequencing data and detection of variations were done by the GS Reference Mapper. An average depth was × 23.3.

The genotypes determined by the GS Junior System were confirmed by direct sequencing using an ABI Prism 3100 Genetic Analyzer (Applied Biosystems). Primers used for the direct sequencing are listed in Table S2. The detected variations were genotyped in additional 96 individuals, and the LD status was examined in the total 108 individuals.

### Transfection and reporter assay

Jurkat T cells were cultured in RPMI 1640 medium supplemented with 10% fetal bovine serum, penicillin (100 U/ml) and streptomycin (0.1 mg/ml) at 37°C in a humidified atmosphere of 5% CO_2_.

*IRF2* region, including the promoter region up to −918 bp, exon 1 and intron 1 encompassing the SNPs rs66801661 and rs62339994, was amplified using primers 5′-GCTAGCCTCGAGGATGCGTGGGCTGTGACTTACTG-3′ and 5′-AGGCCAGATCTTGATGAAAGCCACCTCGCTCTTCT-3′, from the genomic DNA of an individual homozygous for rs66801661G and rs62339994A. To generate a construct termed pGL4-*IRF2* G-A, the PCR product was cloned into the EcoR V site of a firefly luciferase reporter vector, pGL4.10 (Promega, Madison, WI), using GeneArt Seamless Cloning and Assembly kit (Life Technologies, Carlsbad, CA). pGL4-*IRF2* G-G and A-A constructs were generated from the pGL4-*IRF2* G-A by site-directed mutagenesis using PrimeSTAR Mutagenesis Basal Kit (Takara Bio, Inc., Otsu, Japan). Primers used in the mutagenesis were as follows: 5′-GCAGCCTGGGCAGGGCGCCAGAGCCG-3′ and 5′-CCCTGCCCAGGCTGCCCGCCTCCTTC-3′ for pGL4-*IRF2* G-G, and 5′-AGGAGGGAAGCGGGTCCTTCTCGGTG-3′ and 5′-ACCCGCTTCCCTCCTTCGGATCCGGT-3′ for pGL4-*IRF2* A-A.

Transfection was performed using X-tremeGENE HP DNA Transfection Reagent (Roche Applied Science). 1 µg of the pGL4-*IRF2* vectors (G-A, G-G or A-A), 0.1 µg of the pGL4.74 control vector containing *Renilla* luciferase (Promega) and 3 µl of the X-tremeGENE HP reagent were preincubated in 100 µl of Opti-MEM and then added to the cells. Twenty four hours after transfection, the Jurkat T cells were cultured with or without 1000 U/ml of IFNγ (PeproTech Inc., Rocky Hill, NJ) for another 24 hours. Firefly and *Renilla* luciferase activities were measured with the Dual-Luciferase Reporter Assay System (Promega) on the TD-20/20 Luminometer (Turner BioSystems, Sunnyvale, CA). Firefly luciferase activities were normalized to *Renilla* luciferase activities.

### Statistical analysis

Association analysis was carried out by chi-square test using 2×2 contingency tables under the allelic, dominant and recessive models. Correction for multiple testing was performed by Bonferroni correction. The corrected P-values (Pc) were calculated by multiplying by 48 (the number of SNPs analyzed in this study). LD was analyzed using Haploview version 4.0 software (Broad Institute, Cambridge, MA). To evaluate an independent effect of each SNP, conditional logistic regression analysis was employed on the associated SNPs under the additive model. Haplotype association was tested by permutation test using the SNPAlyze software (DYNACOM Co., Ltd., Chiba, Japan). Power calculation based on the sample size of this study was performed with the PS (Power and Sample Size Calculation) program (Table S3). Statistical difference in the luciferase activities was analyzed by one-way ANOVA followed by Tukey's test.

## Results

### Association study of the *IRF2* tag SNPs with SLE

**Figure 1:**
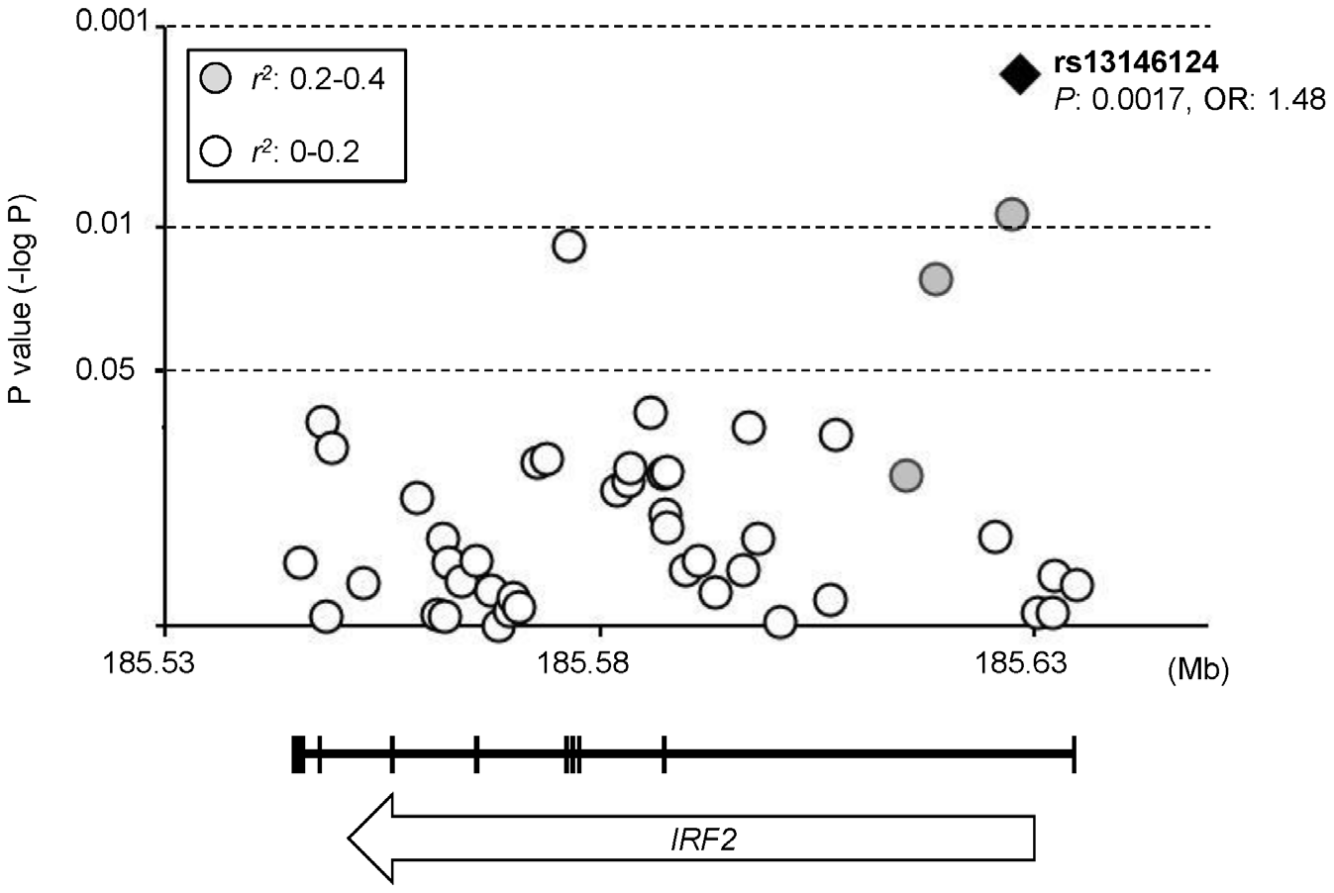
Association of *IRF2* tag SNPs with SLE. P values for differences in allele frequencies were calculated by chi-square test. The -log_10_P value for each SNP is shown. Grey (*r^2^*: 0.2–0.4) and white (*r^2^*: 0–0.2) dots represent pairwise *r^2^* values between rs13146124 and the other tag SNPs. OR: odds ratio.

The *IRF2* gene, spanning approximately 87 kb, is located on chromosome 4q34.1-q35.1 and consists of nine exons. Forty six tag SNPs which capture 100 SNPs in the *IRF2* region (minor allele frequency ≥0.05) with *r^2^*≥0.8 were selected, and a case-control study for the tag SNPs was conducted on the discovery set which consists of 501 Japanese patients with SLE and 551 healthy Japanese controls. P values for allelic association of the tag SNPs are shown in Fig. 1. Departure from Hardy-Weinberg equilibrium was not observed at a significance level of α = 0.01 in the healthy controls. An intronic SNP, rs13146124, was most significantly associated with SLE (P = 0.0017, odds ratio [OR] 1.48, 95% confidence interval [CI] 1.16–1.89). Subsequently, the association of rs13146124 was tested in all cases and healthy individuals. As shown in Table 1, the association was prominent under the dominant model for the minor allele (rs13146124: P = 5.4×10^−4^, OR 1.48, 95% [CI] 1.18–1.85). The difference was also significant when only the female cases and controls were compared (P = 1.5×10^−4^, OR 1.66, 95% CI 1.28–2.15).

**Table 1:** Association of *IRF2* SNPs with SLE.

		Genotype			Minor allele	Allele model		Dominant model	
						P	OR (95%CI)	P	OR (95%CI)
rs13146124		T/T	C/T	C/C	T				
All	SLE	20(2.4)	227(27.2)	587(70.4)	267(16.0)	0.0011	1.39(1.14–1.69)	5.4×10^−4^	1.48(1.18–1.85)
	controls^*^	16(2.0)	165(20.2)	636(77.8)	197(12.1)				
Female	SLE	20(2.6)	211(27.5)	537(69.9)	251(16.3)	5.4×10^−4^	1.51(1.20–1.90)	1.5×10^−4^	1.66(1.28–2.15)
	controls^*^	12(2.3)	95(18.3)	412(79.4)	119(11.5)				
rs66801661		A/A	G/A	G/G	A				
All	SLE	9(1.1)	152(18.2)	673(80.7)	170(10.2)	7.7×10^−4†^	1.53(1.19–1.96)	0.0014	1.54(1.18–2.00)
	controls^*^	3(0.4)	107(13.1)	707(86.5)	113(6.9)				
Female	SLE	9(1.2)	141(18.4)	618(80.5)	159(10.4)	0.0039	1.53(1.15–2.03)	0.0063	1.53(1.13–2.08)
	controls^*^	2(0.4)	69(13.3)	448(86.3)	73(7.0)				
rs62339994		A/A	G/A	G/G	A				
All	SLE	19(2.3)	222(26.6)	593(71.1)	260(15.6)	0.0016	1.38(1.13–1.68)	9.0×10^−4‡^	1.46(1.17–1.82)
	controls^*^	15(1.8)	163(20.0)	639(78.2)	193(11.8)				
Female	SLE	19(2.5)	206(26.8)	543(70.7)	244(15.9)	7.3×10^−4^	1.50(1.19–1.90)	1.9×10^−4^	1.65(1.27–2.15)
	controls^*1^	12(2.3)	92(17.7)	415(80.0)	116(11.2)				

OR: odds ratio, CI: confidence interval. Frequencies are shown in parentheses (%). Association was tested by chi-square analysis using 2×2 contingency tables. *Departure from Hardy-Weinberg equilibrium was not observed (P>0.01). ^†^Pc = 0.037 and ^‡^Pc = 0.043 after Bonferroni correction.

### Resequencing

To examine whether rs13146124 by itself contributes to susceptibility to SLE or is a proxy for another functional polymorphism, resequencing of the regions covering all exons, the intron 1 region encompassing rs13146124, and the promoter region up to 5 kb upstream of *IRF2* gene was performed by next-generation sequencing. Six individuals homozygous for the risk allele rs13146124T and six homozygous for the non-risk rs13146124C were resequenced.

**Figure 2:**
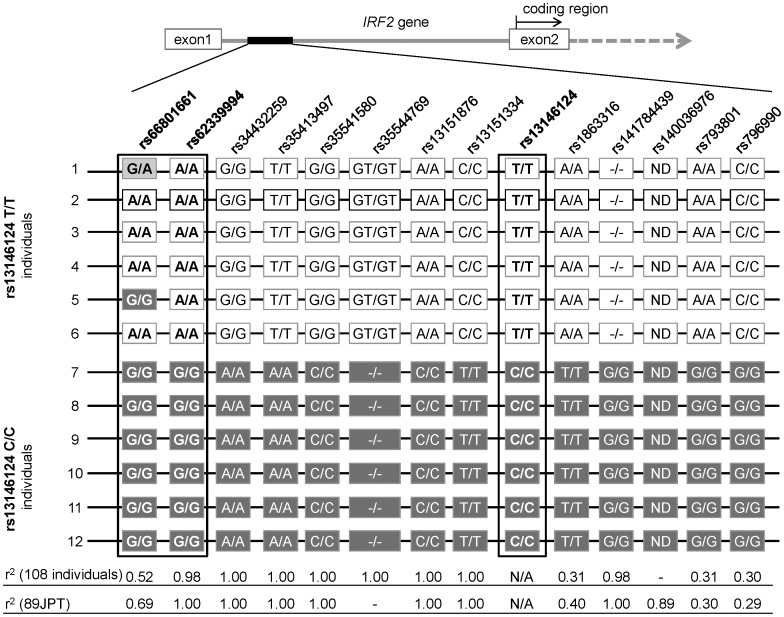
Detection of *IRF2* SNPs in linkage disequilibrium with rs13146124. To find *IRF2* SNPs in linkage disequilibrium with rs13146124, resequencing was performed for 12 individuals (six with rs13146124T/T and six with rs13146124C/C genotype). The color of each square represents the genotype of each SNP. Homozygote for the minor allele, heterozygote, and homozygote for the major allele is indicated by white, light grey and dark grey, respectively. Pairwise *r^2^* values between rs13146124 and each SNP, calculated from 108 individuals genotyped in this study and in 89 HapMap JPT on the 1000 Genomes database, are shown. rs35544769 and rs140036976 was observed only by the resequencing using the GS Junior System and the 1000 Genomes database, respectively. ND: not detected, N/A: not applicable.

This analysis detected 10 SNPs including tag SNPs, rs1863316 and rs793801, and 2 indels which appeared to be in LD with rs13146124 (Fig. 2). All of them were located in intron 1, and none were found in the coding or untranslated region (UTR) of *IRF2*. Except for rs1863316 and rs793801, genotype data of the polymorphisms was not available from the HapMap database. Therefore, the genotypes of these polymorphisms were determined in additional 96 individuals by Sanger sequencing, and their pairwise association was examined in the total 108 individuals (18 with rs13146124T/T, 52 with C/T, and 38 with C/C genotype).

Many of them were strongly associated with rs13146124 (*r^2^* = 0.98–1.00), except for rs66801661, rs1863316, rs793801 and rs796990, which exhibited moderate association (*r^2^* = 0.30–0.52) (Fig. 2).

To further examine a possibility that a causative SNP might exist in the region that was not analyzed by resequencing, we employed the 1000 Genomes database (http://www.1000genomes.org/) and calculated LD between the variations in the whole *IRF2* gene and promoter region up to 17 kb upstream using genotype data in 89 HapMap JPT individuals. rs140036976, listed in the 1000 Genomes database and in LD with rs13146124 (*r^2^* = 0.89), was not detected by resequencing. On the other hand, rs35544769, which was detected by resequencing, was not listed in the 1000 Genomes database. Except for such differences, *r^2^* values detected in the 1000 Genomes database were generally well in accordance with those obtained by resequencing (Fig. 2).

### Association study of the SNPs detected by resequencing

We next investigated whether the association of rs13146124 could be explained by primary association with any of the SNPs which exhibited LD with rs13146124. rs1863316 and rs793801 were already examined as tag SNPs, and rs796990 was tagged by rs793801 (*r^2^* = 0.97). To elucidate potentially functional SNPs, we utilized the ENCODE data (http://genome.ucsc.edu/ENCODE/). Among the polymorphisms which were in LD with rs13146124, rs62339994 and rs66801661 were considered most likely to be involved in transcriptional activity, because the regions containing rs62339994 and rs66801661 were hypersensitive to DNaseI in a number of cell types including immune cells such as T cells, B cells and monocytes (Fig. S2). Therefore, we examined the association of rs62339994 and rs66801661 in the discovery set as well as additional cases and controls, which made the total number of subjects 834 SLE and 817 controls. This analysis demonstrated that, in addition to rs62339994 which is in near absolute LD with rs13146124 (*r^2^* = 0.95), rs66801661A was also significantly associated with SLE (P = 7.7×10^−4^, OR 1.53, 95%CI 1.19–1.96). The association of rs66801661 and rs62339994 remained significant after correction for multiple testing by Bonferroni correction (rs66801661: Pc = 0.037, under the allele model, rs62339994: Pc = 0.043, under the dominant model). In addition, the significance remained when only female cases and controls were compared (rs66801661: P = 0.0039, OR 1.53, 95%CI 1.15–2.03, under the allele model, rs62339994: P = 1.9×10^−4^, OR 1.65, 95%CI 1.27–2.15, under the dominant model). When the association of rs62339994 was conditioned on rs66801661, the association was no longer significant (P_adjusted_ = 0.33) (Table 2). Similarly, the association of rs66801661 was also eliminated by conditioning on rs62339994 (P_adjusted_ = 0.11). Thus, due to LD (*r^2^* = 0.50), it was not possible to determine which of the two SNPs plays the primary role.

**Table 2:** Conditional logistic regression analysis of *IRF2* SNPs associated with SLE.

*IRF2* SNP	risk allele	P*	OR* (95%CI)	P_adjusted_ and OR_adjusted_ (95%CI)† when conditioned on:			
				rs66801661		rs62339994	
rs66801661	A	8.4×10^−4^	1.53 (1.19–1.96)	N/A		0.11	1.34 (0.93–1.93)
rs62339994	A	0.0018	1.37 (1.13–1.68)	0.33	1.16 (0.87–1.55)	N/A	

OR: odds ratio, CI: confidence interval, N/A: not applicable.

^*^P value, OR, and 95%CI calculated by logistic regression analysis under the additive model for each risk allele.

† P value, OR, and 95%CI adjusted for each SNP by conditional logistic regression analysis.

### Haplotype analysis

Then we examined the association of the haplotypes constituted by rs66801661 and rs62339994. The haplotype containing both of the risk alleles (haplotype C), was significantly increased in SLE (permutation P = 9.9×10^−4^) (Table 3). On the other hand, the haplotype constituted by both of the non-risk alleles (haplotype A) was significantly decreased (permutation P = 0.0020).

**Table 3:** Association of *IRF2* haplotypes with SLE.

Haplotype	rs66801661–rs62339994	SLE	Controls	Permutation P-value	Effect
A	G-G	84.4%	88.1%	0.0020	protective
B	G-A	5.8%	5.2%	0.42	neutral
C	A-A	9.8%	6.7%	9.9×10^−4^	risk

P value was calculated by permutation test (100,000 permutations) using SNPAlyze software.

### Association study of *IRF2* with SLE subsets

We next examined association of the *IRF2* SNPs with clinical subsets of SLE. The patients were classified according to presence of renal disorder, malar rash, discoid rash, anti-double strand (ds) DNA antibodies, anti-Sm antibodies, anti-Ro antibodies or anti-La antibodies. When the association was examined between SLE patients with and without each phenotype (case-only analysis), marginal association of discoid rash with rs62339994 was observed (P = 0.029, OR 0.62, 95%CI 0.40–0.95, Table 4).

**Table 4:** Association of *IRF2* SNPs with clinical subsets of SLE (case-only analysis).

	n		rs66801661		rs62339994	
	present	absent	P	OR (95%CI)	P	OR (95%CI)
renal disorder	392	401	0.19	1.25(0.90–1.73)	0.69	1.06(0.80–1.39)
malar rash	334	234	0.24	0.79(0.53–1.17)	0.19	0.80(0.58–1.12)
discoid rash	129	433	0.41	0.81(0.50–1.33)	0.029	0.62(0.40–0.95)
anti-dsDNA	645	140	0.34	1.25(0.79–1.96)	0.49	0.88(0.62–1.26)
anti-Sm	252	514	0.55	0.90(0.63–1.28)	0.16	0.80(0.59–1.09)
anti-Ro	346	267	0.96	1.01(0.69–1.48)	0.96	1.01(0.73–1.38)
anti-La	80	502	0.92	0.97(0.56–1.69)	0.93	1.02(0.65–1.62)

SLE patients with and without each clinical phenotype were compared. OR: odds ratio, CI: confidence interval.

P value, OR, and 95%CI were calculated under the allele model.

### Association of *IRF2* SNPs with transcriptional activity

**Figure 3:**
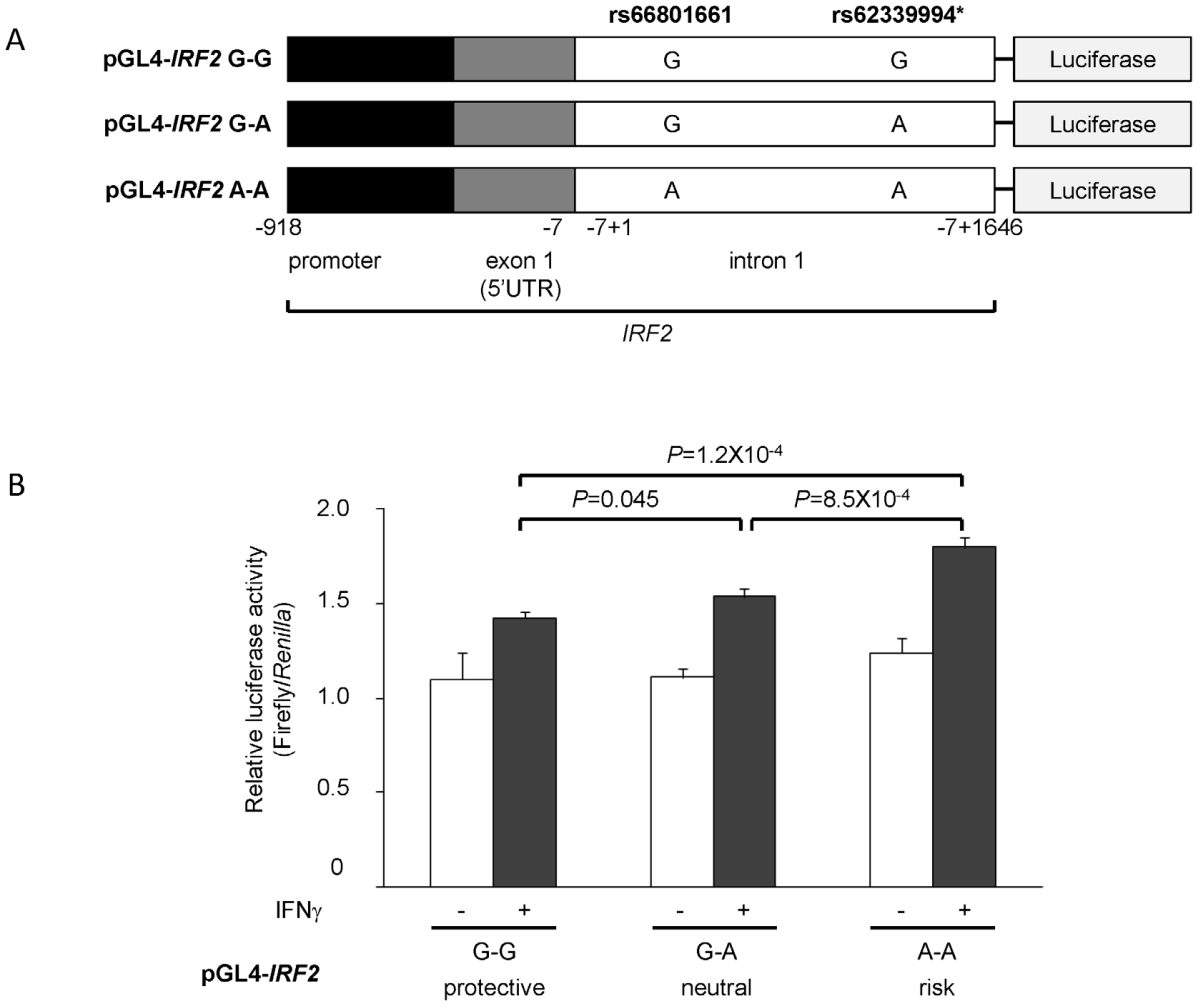
Effect of *IRF2* SNPs on the transcriptional activity of *IRF2.* A. Constructs used in this study. *IRF2* region containing the promoter region, exon 1 and intron 1 encompassing rs66801661 and rs62339994 was inserted upstream of the luciferase gene in pGL4.10. It should be noted that the translation start site of *IRF2* is located within exon 2; therefore, the entire exon 1 constitutes 5′UTR. *rs62339994 is in near absolute LD with rs13146124 (Fig. 2). UTR: untranslated region. **B.** Reporter assay. The constructs were transfected into Jurkat T cells. After transfection, the Jurkat T cells were cultured with or without 1000 U/ml of IFNγ and relative light units (RLU) were measured. P value was calculated by one-way ANOVA followed by Tukey's test. Bars show the mean ± SD of relative luciferase activity (Firefly RLU/*Renilla* RLU) with (shaded) or without (white) stimulation with IFNγ.

Finally, we examined whether rs66801661 and/or rs62339994 influence the transcriptional activity, using reporter assays. A 2.6 kb fragment spanning from the promoter region to the 5′ part of intron 1 encompassing the SNPs rs66801661 and rs62339994 was inserted upstream of the luciferase gene in pGL4.10. Three constructs, pGL4-*IRF2* G-G, G-A, and A-A, corresponding to the naturally-occurring haplotypes A, B and C (Table 3), were prepared to examine the differences in the effect on luciferase activity among the haplotypes (Fig. 3A). The constructs were transfected into Jurkat T cells and the luciferase activities were measured. The experiments were repeated three times with similar results. The representative data are shown in Fig. 3B.

No significant difference was observed when the Jurkat T cells were cultured without stimulation. However, when the Jurkat T cells were stimulated with IFNγ which had previously been shown to induce expression of *IRF2* [24], the luciferase activity was significantly different among the three constructs (one-way ANOVA, P = 1.3×10^−4^). The risk haplotype pGL4-*IRF2* A-A showed the highest (mean ± SD 1.80±0.05), while the protective pGL4-*IRF2* G-G exhibited the lowest luciferase activity (1.42±0.03, A-A vs. G-G: P = 1.2×10^−4^). pGL4-*IRF2* G-A showed moderate luciferase activity in-between of the other two (1.54±0.04). These results suggested that both SNPs contribute to the transcriptional activity.

## Discussion

In this study, through the screening of 46 tag SNPs covering the entire *IRF2* gene, we detected association of rs13146124 located in intron 1. To search for functional polymorphisms that can account for the association of rs13146124, we resequenced all exons, the promoter region and the intron 1 of *IRF2*, and also performed the 1000 Genomes database search. Among the SNPs in LD with rs13146124, rs62339994 and rs66801661 were also associated with SLE. Subsequent reporter assay showed a possibility that rs66801661 and rs62339994 may be the functional SNPs, and that the possession of the risk alleles may result in higher transcriptional activity of *IRF2*. Our observations demonstrated that *IRF2* may contribute to susceptibility to SLE in a Japanese population, which supports the findings by Ramos et al in a European-American population [20], [21]. Unfortunately, their association study was published only in the form of a meeting abstract which does not contain the information on the actual SLE-associated SNP, and we were unable to compare or combine our data with theirs. Nevertheless, association of the same gene both in the European-American and Asian populations provides supporting evidence that *IRF2* may play a role in the genetics of SLE.

In our reporter assay, the differential effects of the *IRF2* SNPs were observed under IFNγ stimulation, suggesting that transcription factors activated by IFNγ may contribute to the difference in the transcriptional activity. Among the transcription factors predicted to bind to the *IRF2* SNP sites, NF-κB has been reported to be activated by IFNγ [25]. The crucial role of NF-κB pathway in the development of SLE is well recognized [26], [27].

When association of the *IRF2* SNPs with *IRF2* expression was analyzed using GENEVAR database (http://www.sanger.ac.uk/resources/software/genevar/), significant association between rs13146124 and *IRF2* mRNA level was not observed in B cell lines established from the HapMap subjects. Data for rs66801661 and rs62339994 were not available in the GENEVAR database. No information on these 3 SNPs were found in eqtl.uchicago.edu (http://eqtl.uchicago.edu) database and GTEX portal database (http://www.gtexportal.org). In our study, elevated transcription of luciferase gene was observed in the Jurkat T cells after stimulation with IFNγ. Differences in the cell types and the stimulation conditions might explain the difference of the results. In fact, recent studies showed that the effect of the polymorphisms on the regulation of gene expression is different according to the cell types [28].

IRF2 has been shown to have various functions in immune responses. IRF2 attenuates type I IFN responses [12]. *Irf2* deficient mice developed an inflammatory skin disease similar to psoriasis, which was thought to be attributable to enhanced type I IFN signaling [29]. Thus, it was somewhat unexpected that the *IRF2* risk haplotype identified in this study was associated with higher transcriptional activity. It should be noted that IFN signature is not observed in all SLE patients, and only about a half of the patients show elevated serum IFNα activity [30]. In the future, it will be interesting to collectively examine the association of genotypes of type I IFN pathway genes including *IRF2*, with IFN signature.

On the other hand, IRF2 is required for Th1 differentiation, and lack of *irf2* has been shown to result in impaired IL-12 production and a defect in Th1 cell differentiation [31]. Furthermore, IRF2 has been shown to be required for NK cell development [31].

Of interest, *STAT4*, one of the strongest susceptibility genes to SLE identified so far, is essential for Th1 differentiation, and the risk alleles have been suggested to be associated with gain of function of STAT4 [1]–[3], [32], [33]. Thus, it is probable that the *IRF2* SNPs may contribute to SLE by pathways involving Th1 and/or NK cells.

Thus far, some studies demonstrated important roles of IFNγ as well as Th1 responses in SLE. Enhanced IFNγ was observed in human SLE [34], and increased expression of IFNγ-inducible genes has been shown in SLE monocytes [35]. In the case of murine lupus, contribution of IFNγ is well known. In NZBxW F1 mice, development of autoantibody and nephritis were prevented by IFNγ receptor deletion [36], and treatment with anti-IFNγ delayed onset [37]. In humans, Th1/Th2 balance was reported to be different among the types of lupus nephritis, and some data suggested that Th1 was dominant in proliferative lupus nephritis [38], although this remains controversial [39]. These observations imply that not only type I IFN, but also IFNγ and Th1 play a significant role in SLE.

Our group previously reported association of SNPs in *SPI1* gene, which encodes a transcription factor PU.1, with SLE [40]. Interestingly, PU.1 has been shown to interact with IRF2 and IRF8 in the transcriptional activation of Nf1, a Ras-GAP protein that regulates cytokine-induced proliferation of myeloid cells [41]. Because IRF8 has already been associated with susceptibility to SLE [6], [16], the present study implicates that all of these three genes may be associated with SLE. This suggests that the molecular complex involving these three molecules may play a substantial role in SLE.

This study suffers from several limitations. With respect to the detected SNPs, independent replication studies are required. Because a small number of prospective SLE patients might be present in the control group, the analysis of this study could be conservative. Regarding the analysis of SLE subsets, the power to detect significant association is compromised due to the small number of subjects (Table S3).

## Conclusions

Association of *IRF2* with SLE was observed in a Japanese population. Functional analysis demonstrated that the SLE risk haplotype was associated with transcriptional activation of *IRF2*. These findings support the previous reports that suggested the role of *IRF2* in the genetic risk for SLE in a European-American population.

## Supporting Information

Figure S1 **Flow diagram of this study.**
(TIF)

Figure S2 ***IRF2*** **region encompassing rs66801661 and rs62339994 in the UCSC Genome Browser (** http://genome.ucsc.edu/ **).** Digital DNaseI hypersensitivity clusters and transcription factor ChIP-seq data in *IRF2* region surrounding rs66801661 and rs62339994 are shown. In 123 out of 125 assayed cell types, the region containing rs62339994 showed sensitivity to DNase I. With respect to rs66801661, 78 cell types showed DNase I sensitivity. These cells include immune cells such as T cells, B cells, and monocytes.
(TIF)

Table S1 **Primers used for the resequencing of** ***IRF2.***
(DOC)

Table S2 **Primers used for genotyping by Sanger sequencing.**
(DOC)

Table S3 **Power calculation under the allele model.**
(DOC)
