# Supplementary figures and images for: Association of Functional Polymorphisms in Interferon Regulatory Factor 2 (IRF2) with Susceptibility to Systemic Lupus Erythematosus: A Case-Control Association Study
Source: PLoS One. 2014 Oct 6;9(10):e109764. doi: 10.1371/journal.pone.0109764 (PMC4186848; doi:10.1371/journal.pone.0109764)

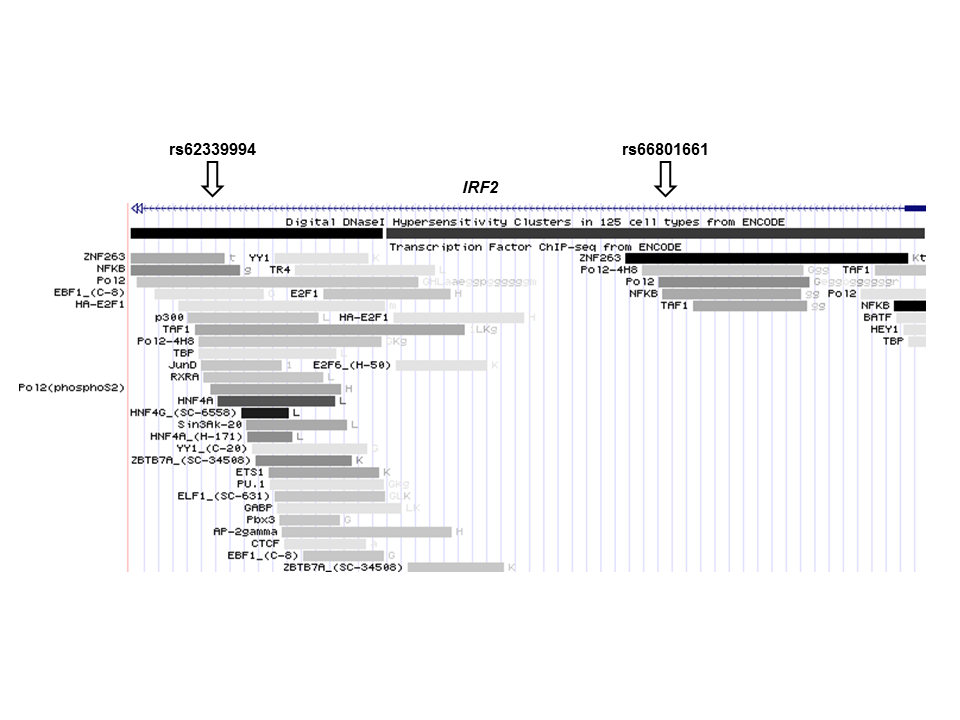

Supplement: Figure S2 — IRF2 region encompassing rs66801661 and rs62339994 in the UCSC Genome Browser ( http://genome.ucsc.edu/ ). Digital DNaseI hypersensitivity clusters and transcription factor ChIP-seq data in IRF2 region surrounding rs66801661 and rs62339994 are shown. In 123 out of 125 assayed cell types, the region containing rs62339994 showed sensitivity to DNase I. With respect to rs66801661, 78 cell types showed DNase I sensitivity. These cells include immune cells such as T cells, B cells, and monocytes. (TIF) [file pone.0109764.s002.tif]
