# Supplementary material for: Association of Functional Polymorphisms in Interferon Regulatory Factor 2 (IRF2) with Susceptibility to Systemic Lupus Erythematosus: A Case-Control Association Study
Source: PLoS One. 2014 Oct 6;9(10):e109764. doi: 10.1371/journal.pone.0109764 (PMC4186848; doi:10.1371/journal.pone.0109764)
Supplement: Table S1 — Primers used for the resequencing of IRF2. (DOC) [file pone.0109764.s003.doc]

Table S1. Primers used for the resequencing of *IRF2.*

|  | Forward primer | Reverse primer | Product length |
| --- | --- | --- | --- |
| 1 | 5’-CAGAACAGGGCAGAATGAGTATCAC-3’ | 5’-CCTCGAGAGAAACACACACAGTCA-3’ | 5498 bp |
| 2 | 5’-ATACCTTGCGGAATTGTATTGGT-3’ | 5’-TCAAATCTAAAAGGTCCCGTGTAA-3’ | 5609 bp |
| 3 | 5’-CTCCAATCACTTCCTCCTTTGGA-3’ | 5’-CCGTAGCAATCCAAAAACGTCTC-3’ | 5233 bp |
| 4 | 5’-CCTTTACCAGACCCCTGTTGGAG-3’ | 5’-TTCCGGCCAGTCAGAAGTCAG-3’ | 5769 bp |
| 5 | 5’-TTTGTCTTGGTGCTTTGCTTTACC-3’ | 5’-TGGACACACAGATAGGGCTTCATTA-3’ | 3911 bp |
| 6 | 5’-TTGCACATTTCAAGAGGGTCCAT-3’ | 5’-GAAATGGGAGGGGCTATTGATGA-3’ | 4716 bp |
| 7 | 5’-GAAGCGAGACCACAGGAACTAAT-3’ | 5’-TAAGGGGTGAACAACTCAGCTTC-3’ | 3973 bp |
| 8 | 5’-ACCTGAATACTTTGCCGATTGGT-3’ | 5’-GTAAGAGTCCAAAACGCATTTGCT-3’ | 4064 bp |
| 9 | 5’-GATTCCATTAATCCAGCCTGTTGTG-3’ | 5’-AAACCAGTGGCGTGGCTAAAATACT-3’ | 4789 bp |
