# Supplementary material for: Association of Functional Polymorphisms in Interferon Regulatory Factor 2 (IRF2) with Susceptibility to Systemic Lupus Erythematosus: A Case-Control Association Study
Source: PLoS One. 2014 Oct 6;9(10):e109764. doi: 10.1371/journal.pone.0109764 (PMC4186848; doi:10.1371/journal.pone.0109764)
Supplement: Table S2 — Primers used for genotyping by Sanger sequencing. (DOC) [file pone.0109764.s004.doc]

Table S2. Primers used for genotyping by Sanger sequencing.

|  | Forward primer | Reverse primer | Genotyped SNPs |
| --- | --- | --- | --- |
| 1 | 5’-GGATTTGCAGATGCGGAGA-3’ | 5’-GCCCCAGCAAGTTTCCTCT-3’ | rs66801661 |
| 2 | 5’-GAAGCCGTTCCAAGTTCCAA-3’ | 5’-CCTCGCTCTTCTGAGCTACC-3’ | rs62339994 |
| 3 | 5’-TGTCTGCGTTAAGCCCTTTT-3’ | 5’-TCCAAGAGTTCTTGTCTGTTACG-3’ | rs34432259, rs35413497, rs35541580 |
| 4 | 5’-TTTCCATATCATTTGGTCTCCA-3’ | 5’-GGCCAGATAATCATGACTGGAT-3’ | rs35544769 |
| 5 | 5’-CACATGAGCCAAGATGGTAAAGC-3’ | 5’-GTGTGCACTGGGCTGATTGT-3’ | rs13151876, rs13151334 |
| 6 | 5’-ATGTCCCAACCTCACCAAAA-3’ | 5’-CTAATATGTCATCTCTGCCCTCA-3’ | rs141784439 |
| 7 | 5’-TTTAAAATCGTTGCCCCAAG-3’ | 5’-GACAGCAGGAGAAGCCATTC-3’ | rs796990 |
