# Supplementary material for: Association of Functional Polymorphisms in Interferon Regulatory Factor 2 (IRF2) with Susceptibility to Systemic Lupus Erythematosus: A Case-Control Association Study
Source: PLoS One. 2014 Oct 6;9(10):e109764. doi: 10.1371/journal.pone.0109764 (PMC4186848; doi:10.1371/journal.pone.0109764)
Supplement: Table S3 — Power calculation under the allele model. (DOC) [file pone.0109764.s005.doc]

Table S3. Power calculation under the allele model.

| phenotype | Sample size | |  | rs66801661 | | |  | rs62339994 | | |  | rs13146124 | | |
| --- | --- | --- | --- | --- | --- | --- | --- | --- | --- | --- | --- | --- | --- | --- |
|  | +* | -* |  | 1.3 | 1.5 | 1.7 |  | 1.3 | 1.5 | 1.7 |  | 1.3 | 1.5 | 1.7 |
| all SLE | 834 | 817 |  | 0.523 | 0.895 | 0.991 |  | 0.723 | 0.981 | 1 |  | 0.73 | 0.983 | 1 |
| renal disorder | 392 | 401 |  | 0.353 | 0.709 | 0.92 |  | 0.492 | 0.866 | 0.983 |  | N/A | N/A | N/A |
| malar rash | 334 | 234 |  | 0.293 | 0.615 | 0.857 |  | 0.381 | 0.748 | 0.94 |  | N/A | N/A | N/A |
| discoid rash | 129 | 433 |  | 0.241 | 0.498 | 0.732 |  | 0.312 | 0.628 | 0.854 |  | N/A | N/A | N/A |
| anti-dsDNA | 645 | 140 |  | 0.189 | 0.419 | 0.671 |  | 0.313 | 0.658 | 0.893 |  | N/A | N/A | N/A |
| anti-Sm | 252 | 514 |  | 0.358 | 0.706 | 0.913 |  | 0.467 | 0.836 | 0.973 |  | N/A | N/A | N/A |
| anti-Ro | 346 | 267 |  | 0.289 | 0.609 | 0.852 |  | 0.388 | 0.758 | 0.945 |  | N/A | N/A | N/A |
| anti-La | 80 | 502 |  | 0.194 | 0.392 | 0.6 |  | 0.238 | 0.487 | 0.716 |  | N/A | N/A | N/A |

Power calculation based on the sample size of this study was carried out using the PS (Power and Sample Size Calculation) program. The power was calculated for the odds ratio of 1.3, 1.5 and 1.7.

*Sample size: For the analysis of “all SLE”, +: patients with SLE, – : healthy controls.

For the analysis of each subphenotype, +: patients with each subphenotype, -: patients without each subphenotype

N/A: not applicable
